# Spinal miRNA-124 regulates synaptopodin and nociception in an animal model of bone cancer pain

**DOI:** 10.1038/s41598-017-10224-1

**Published:** 2017-09-08

**Authors:** Sara Elramah, María José López-González, Matthieu Bastide, Florence Dixmérias, Olivier Roca-Lapirot, Anne-Cécile Wielanek-Bachelet, Anne Vital, Thierry Leste-Lasserre, Alexandre Brochard, Marc Landry, Alexandre Favereaux

**Affiliations:** 1 Bordeaux University, Bordeaux, France; 2 CNRS UMR 5297 « Central mechanisms of pain sensitization », Institut Interdisciplinaire de Neuroscience, 146 rue Léo Saignat, Bordeaux Cedex, 33077 France; 3 Department of Anesthesia and Pain, Institut Bergonié, Bordeaux, France; 4 Service de Neurologie Groupe hospitalier Sud Hôpital Haut-Lévêque, Avenue de Magellan, Pessac Cedex, 33604 France; 5 Univ. Bordeaux, Institut des Maladies Neurodégénératives, UMR 5293, Bordeaux, F-33000 France; 6 INSERM U862 « Physiopathologie de l’addiction », Institut François Magendie, 146 rue Léo Saignat, Bordeaux Cedex, 33077 France

## Abstract

Strong breakthrough pain is one of the most disabling symptoms of cancer since it affects up to 90% of cancer patients and is often refractory to treatments. Alteration in gene expression is a known mechanism of cancer pain in which microRNAs (miRNAs), a class of non-coding regulatory RNAs, play a crucial role. Here, in a mouse model of cancer pain, we show that miR-124 is down-regulated in the spinal cord, the first relay of the pain signal to the brain. Using *in vitro* and *in vivo* approaches, we demonstrate that miR-124 is an endogenous and specific inhibitor of synaptopodin (Synpo), a key protein for synaptic transmission. In addition, we demonstrate that Synpo is a key component of the nociceptive pathways. Interestingly, miR-124 was down-regulated in the spinal cord in cancer pain conditions, leading to an up-regulation of Synpo. Furthermore, intrathecal injections of miR-124 mimics in cancerous mice normalized Synpo expression and completely alleviated cancer pain in the early phase of the cancer. Finally, miR-124 was also down-regulated in the cerebrospinal fluid of cancer patients who developed pain, suggesting that miR-124 could be an efficient analgesic drug to treat cancer pain patients.

## Introduction

Spontaneous pain results from the stimulation of a primary nociceptive afferent that makes synapse in the dorsal horn of the spinal cord. Pain information travels to the supra-spinal areas (prefrontal cortex, cingulate, and parietal cortex) via the thalamus for further processing. Chronic pain can arise from long-term sensitization at any point on this pathway. It is characterized by two main components: allodynia, which is pain due to a stimulus that does not usually lead to pain and hyperalgesia, which is increased pain from a stimulus that usually triggers pain. Pain is one of the most frequent and distressing symptoms in the course of cancer^1^. Often it is the first symptom, and up to 90% of patients with advanced cancer stage suffer from disabling cancer pain which is often unresponsive to treatments^2^. Among the different types of cancer inducing disabling pain, bone cancer is one of the most common^3^. Bone cancer can originate from primary or metastatic tumors and the pain associated with it is considered as the most difficult to treat^4^. To evaluate current therapies and develop new ones, better knowledge of the mechanisms underlying bone cancer pain is required^5^. Analysis of bone cancer models has shown that cancer pain can be due to sensitization of primary afferent neurons innervating the affected bone^6^. In addition, central sensitization of the spinal cord neurons which relay the pain information to the brain has been demonstrated^6^. Although there is a correlation between specific neurochemical and cellular reorganization in the spinal cord and cancer-pain behavior^6^, the underlying molecular mechanisms are still poorly understood. Some studies have demonstrated that increased pain levels may result from alterations in the expression of genes involved in nociception, such as dynorphin, substance P and glutamate^5, 6^. Furthermore, it has been shown that the alteration of gene expression in the spinal cord in chronic pain conditions can be caused by microRNAs (miRNAs) (for review see ref. 7). MiRNAs are non-coding RNAs binding to recognition elements in multiple target mRNAs that silence expression via post-transcriptional mechanisms operating as a master switch of the genome^8^. miRNAs are transcribed into a primary-miRNA (pri-miRNA) mainly by RNA polymerase II^9^. Then, pri-miRNA is cleaved by Drosha, a RNase III endonuclease, producing a 60–70 nt stem loop intermediate known as pre-miRNA^10–13^. This pre-miRNA is actively transported from the nucleus to the cytoplasm^14, 15^, then further processed by Dicer, a RNase III endonuclease that cleaves the terminal base pairs and the loop of the pre-miRNA, leaving an imperfectly matching duplex. At this stage, only one strand is finally incorporated into the RNA-induced silencing complex (RISC), the “guide” strand, whereas the other strand called “the passenger” (also called the miRNA* strand) is likely degraded. The interaction between miRNA and target mRNAs occurs at a specific sequence in the 3′ untranslated region (UTR) of the target mRNA. The nucleotide sequence in the target mRNA that is engaged in miRNA binding is called the miRNA Recognition Element (MRE)^16^ or “seed region”^17^. miRNA-RISC-mediated gene inhibition has been demonstrated to arise from three putative mechanisms: (i) site-specific cleavage, (ii) enhanced mRNA decay and (iii) translational inhibition^17^. The mechanism of inhibition by miRNAs is a complex network of interactions between miRNAs and mRNAs. Indeed, a single miRNA can target multiple genes but at the same time, one gene can be targeted by multiple miRNAs^18^.

In 2007, the pioneering study by Bai *et al*. suggested the involvement of miRNAs in the development and/or maintenance of inflammatory pain^19^. Solid proof of this hypothesis was provided in 2010 by Zhao *et al*. They demonstrated the importance of miRNAs in an inflammatory pain model by deleting the Dicer enzyme in the nociceptive neurons of the dorsal root ganglia^20^. Hence, without Dicer, miRNA production in the nociceptive neurons was abolished and resulted in inflammatory pain attenuation. Since then, many other miRNAs have been described as pain regulators in most, if not all, pain models such as sciatic nerve ligation^21^, diabetic neuropathy^22, 23^ and chronic constriction injury^24, 25^. Finally, a recent study revealed the involvement of miRNAs in cancer pain mechanisms in primary sensory neurons^26^ but their role in the regulation of gene expression in the spinal cord remain unknown.

In this report combining genome-wide screening, *in silico* analyses, a behavioral study in a cancer pain mouse model and investigation of patients’ samples, we demonstrate the clinical potential of miR-124 as a cancer-pain analgesic. Indeed, we show that in cancer conditions, miR-124 is down-regulated in the spinal cord, thereby inducing a specific up-regulation Synpo, a key protein for synaptic transmission and pain processing. Intrathecal injections of miR-124 in cancer mice efficiently normalized Synpo expression and alleviated cancer pain. In addition, analysis of patients’ cerebrospinal fluid showed miR-124 to be a cancer-pain biomarker, suggesting its therapeutic potential for pain relief in cancer patients.

## Results

### Histological and behavioral analyses

**Figure 1:**
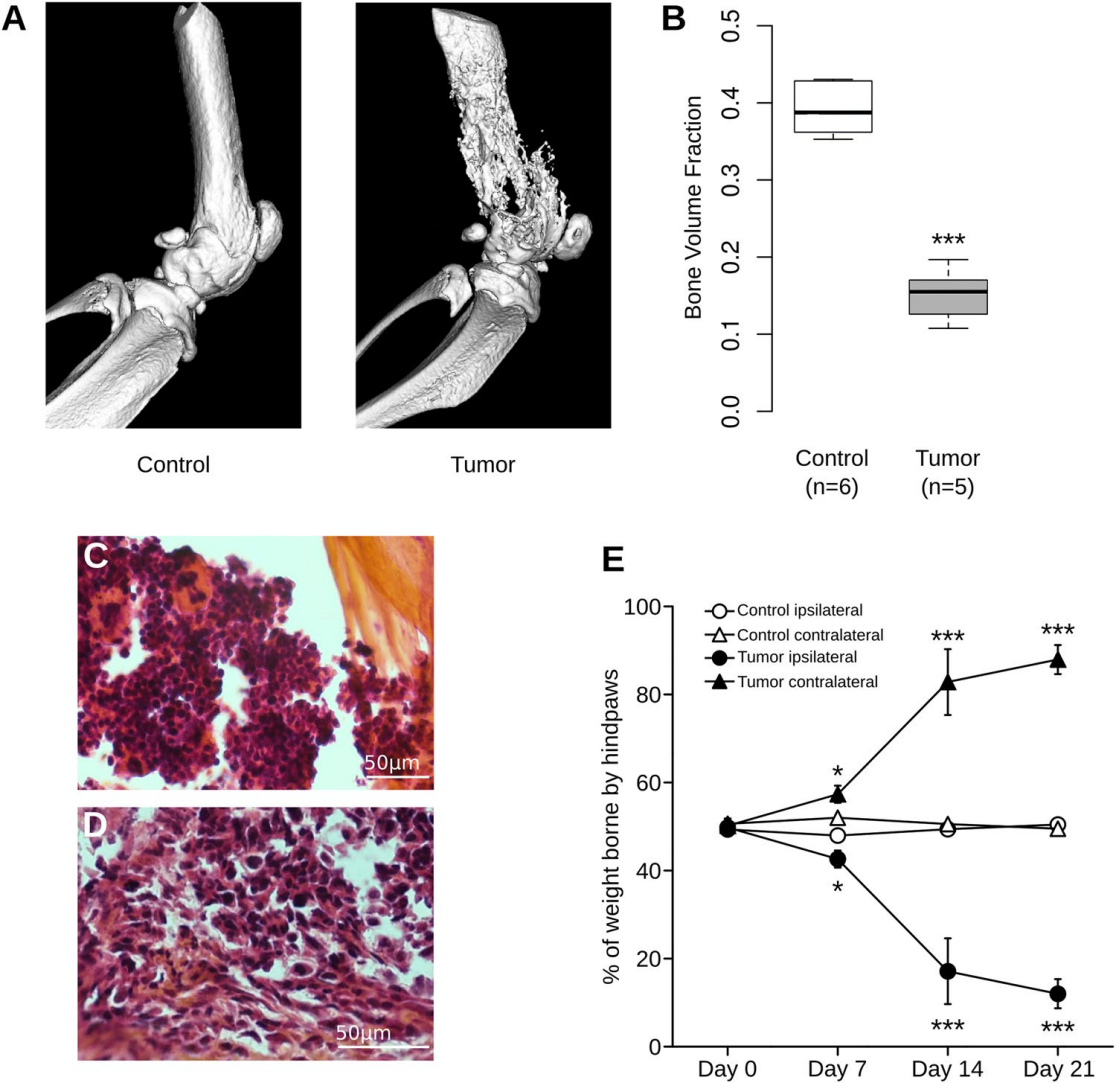
Quantification of bone destruction and assessment of nociceptive state. (**A**) Radiographic images of intact femur 21 days after saline *(left)* or sarcoma cells injection *(right)*, revealing destruction of bone upon tumor development. (**B**) Bone volume quantification shows significant loss of bone density in tumor-injected group at day 21 after surgery (***P < 0.001, Mann-Whitney). (**C**) Hematoxylin-eosin-safran staining in control animals clearly shows normal structure of bone and bone marrow cells. (**D**) In contrast, in mice injected with NCTC 2472 sarcoma cells, there is a clear degradation of bone and replacement of bone marrow by sarcomatous cells. (**E**) Tumor-injected mice show a significant decrease in weight borne by ipsilateral paw compared to control group at days 7, 14 and 21 after surgery. Contralateral paws of tumor-injected group show a significant increase in weight borne at days 7, 14 and 21 after surgery in comparison to control group. Statistical comparison of tumor and control groups performed with two-way ANOVA with repeated measures followed by Bonferroni post-test, *P < 0.05, ***P < 0.001 compared to day 0.

In this study, we used a murine model of bone cancer pain developed by Schwei *et al*.^6^ that consists in injecting osteolytic sarcoma cells into the intramedullary space of the mouse femur. Twenty-one days after injection, 3D high-resolution X-Ray (µCT) images revealed a massive bone resumption in the femur of sarcoma-injected mice in comparison to sham animals which received saline injection (Fig. 1A and B, P < 0.001, Mann-Whitney). The severity of the cancer model was also assessed by classical histology. Hematoxylin-eosin-safran staining clearly showed a normal structure of bone and bone marrow in control animals (Fig. 1C). In contrast, in mice injected with NCTC 2472 sarcoma cells, the bone was clearly degraded and the bone marrow was replaced by sarcomatous cancer cells (Fig. 1D).

In addition to bone remodeling, significant pain-related behavior was observed in mice injected with sarcoma cells compared to sham-injected mice. We used the dynamic weight bearing (DWB) test which is an efficient method to assess cancer pain^27, 28^. The DWB revealed a significant reduction in the weight borne by the tumor-injected paw appearing on day 7 (P < 0.05), increasing on day 14 (P < 0.001) and until the final day of the experiment (day 21, P < 0.001). The decrease was greater than 75% compared to sham animals (Fig. 1E, two-way ANOVA with repeated measures followed by a Bonferroni post-test). The reduction in the weight borne by the ipsilateral paw was compensated by an increase in the weight borne by the contralateral paw (Fig. 1E, P < 0.05). In contrast, the weight was equally borne by the ipsilateral and contralateral paws in the control group. Furthermore, cancerous mice spent more time leaning on the intact paws than on the tumor-bearing paw (Supp Fig. 1, P < 0.01, two-way ANOVA followed by Bonferroni post-test). Additional control experiments consisting in injection of sarcoma cells in the quadriceps did not lead to the development of cancer pain (Supp Fig. 2). Altogether these results demonstrate the development of mechanical allodynia in this mouse model. In addition, the Hargreaves test^29^ has been used to examine paw thermal sensitivity to noxious heat stimuli. The tumor group showed a moderate but significant decrease in paw withdrawal latency by day 21 compared to the control group, indicating that they displayed primary heat hyperalgesia (Supp Fig. 3, P < 0.001, one-way ANOVA with repeated measures followed by Bonferroni post-test).

### mRNA and miRNA screening

We sought to identify the role of miRNAs in cancer-pain mechanisms through the regulation of mRNAs. To achieve this goal, we first screened mRNA and miRNA expression, then did a correlation analysis based on miRNA target prediction algorithms to identify which miRNAs regulate which mRNAs.

**Figure 2:**
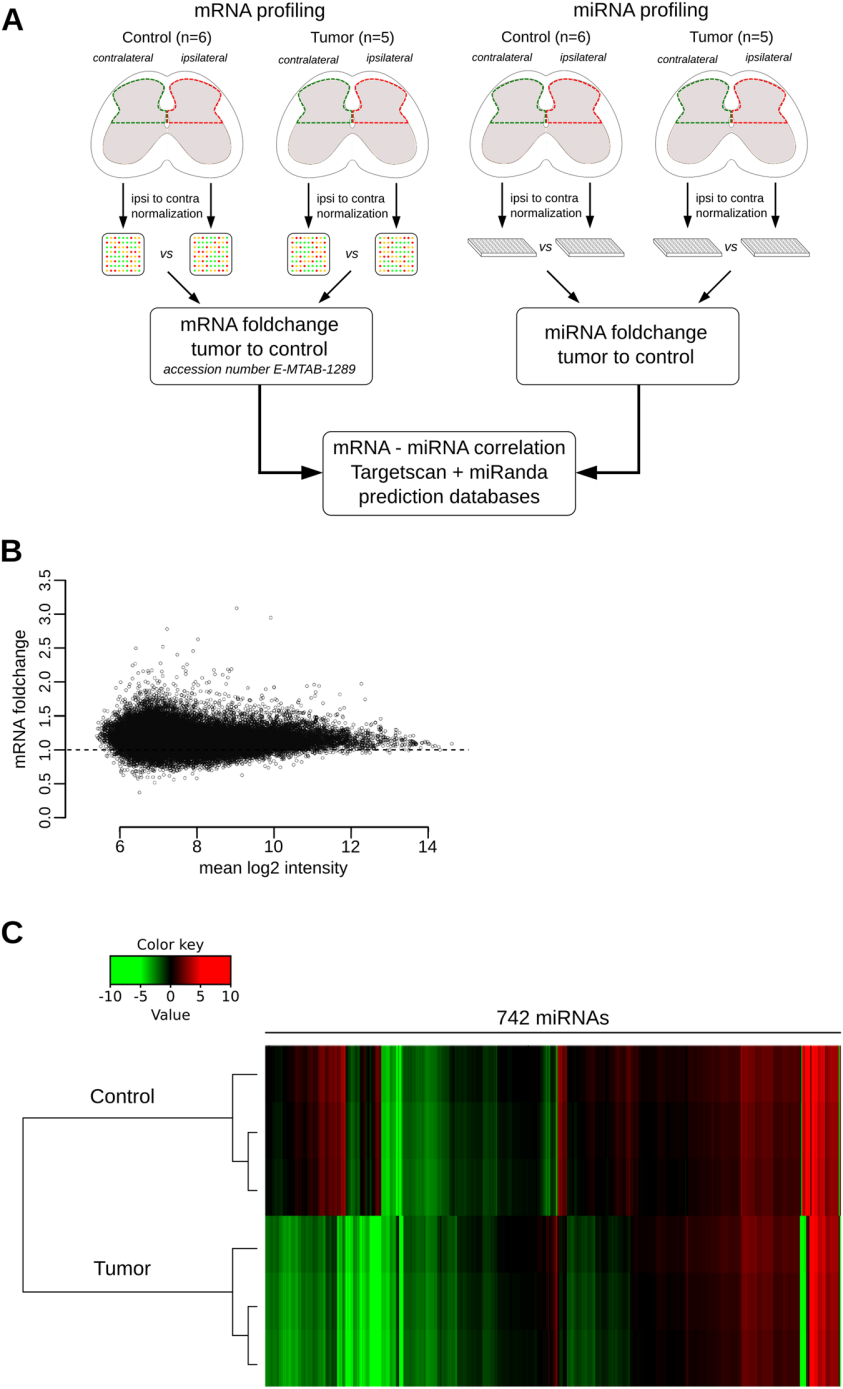
Profiling of mRNAs and miRNAs in the spinal cord of cancerous mice. (**A**) Schematic representation of RNA screening strategy. (**B**) MA plot representing differential expression of mRNAs in bone-cancer-pain condition compared to control condition: 3909 mRNAs differentially expressed. **(C)** Heatmap representation of miRNA expression in cancerous versus naive mice: out of 742 miRNAs tested, 525 miRNAs were differentially expressed with 175 up-regulated (fold change > 2) and 350 down-regulated (fold change < 0.5).

Nociceptive information from the tumor-bearing paws was processed in the lumbar enlargement of the spinal cord, and particularly in the dorsal horns. To profile and correlate the alterations in the expression of mRNAs and miRNAs in the spinal cord of bone-cancer-pain animals, we collected the dorsal horn of the lumbar enlargement from 5 tumor mice and 6 control mice. Both the ipsilateral and contralateral sides of the spinal cord were collected from each mouse and the gene expression of the ipsilateral side was normalized to that of the contralateral side. RNA samples were pooled to create four groups: (i) ipsilateral and (ii) contralateral sides of the dorsal horn of the spinal cord of tumor-bearing mice and (iii) ipsilateral and (iv) contralateral sides of the dorsal horn of the spinal cord of control mice. Pooled RNA samples representing each group were analyzed with microarray and RT-qPCR-based methods to quantify mRNA and miRNA expression, respectively (Fig. 2A). A total of 3909 mRNAs were differentially expressed between the tumor and the control group (data deposited in ArrayExpress, accession number: E-MTAB-1289). Overall, most of the mRNAs were found to be up-regulated in the bone-cancer-pain condition (Fig. 2B). These differentially expressed mRNAs represent genes with a wide range of molecular functions, biological processes and cellular components, as assessed by Gene Ontology term enrichment analysis (Supp Fig. 4).

Interestingly, miRNA screening revealed that 525 out of the 742 miRNAs investigated were dysregulated in the spinal cord, with 175 up-regulated (fold change > 2) and 350 down-regulated (fold change < 0.5) in the cancer-pain condition (Fig. 2C). Since miRNAs exert an inhibitory regulation on mRNA expression, these results suggest that the changes in gene expression observed in the spinal cord in cancer-pain conditions involve a regulation by the miRNA system.

To identify which miRNAs regulate which mRNAs in our large dataset, we developed a software to correlate mRNA and miRNA data in the light of the miRNA-target prediction databases. However, since a single miRNA can target dozens of mRNAs, the result of this anti-correlation analysis is a list of 8670 miRNA-mRNA pairs (Supp Table 1). Such a large number of candidates was obviously too great to run molecular experiments on each mRNA to confirm miRNA targeting. To shorten the candidate list, we searched miRNAs predicted to regulate multiple mRNAs involved in pain transmission. This analysis of our screening highlighted miR-124 for several reasons. First, miR-124 is one of the most regulated miRNAs in the spinal cord in bone-cancer-pain conditions, since its expression was 70% lower than in the control group (Supp Table 1). Second, among miR-124 targets (Supp Table 2), mRNA screening showed that 44 genes involved in neuronal physiology were up-regulated in cancer-pain conditions. Furthermore, miR-124 is one of the most enriched miRNAs of the central nervous system^30^ and is involved in many physiological processes including development^31–35^, plasticity^36, 37^ and pathology^38–40^. Interestingly, miR-124 has been implicated in the establishment and progression of chronic inflammatory and neuropathic pain^41^. Therefore, we focused our analysis on the role of miR-124 in the spinal cord in cancer-pain conditions.

### miR-124 targets identification

**Figure 3:**
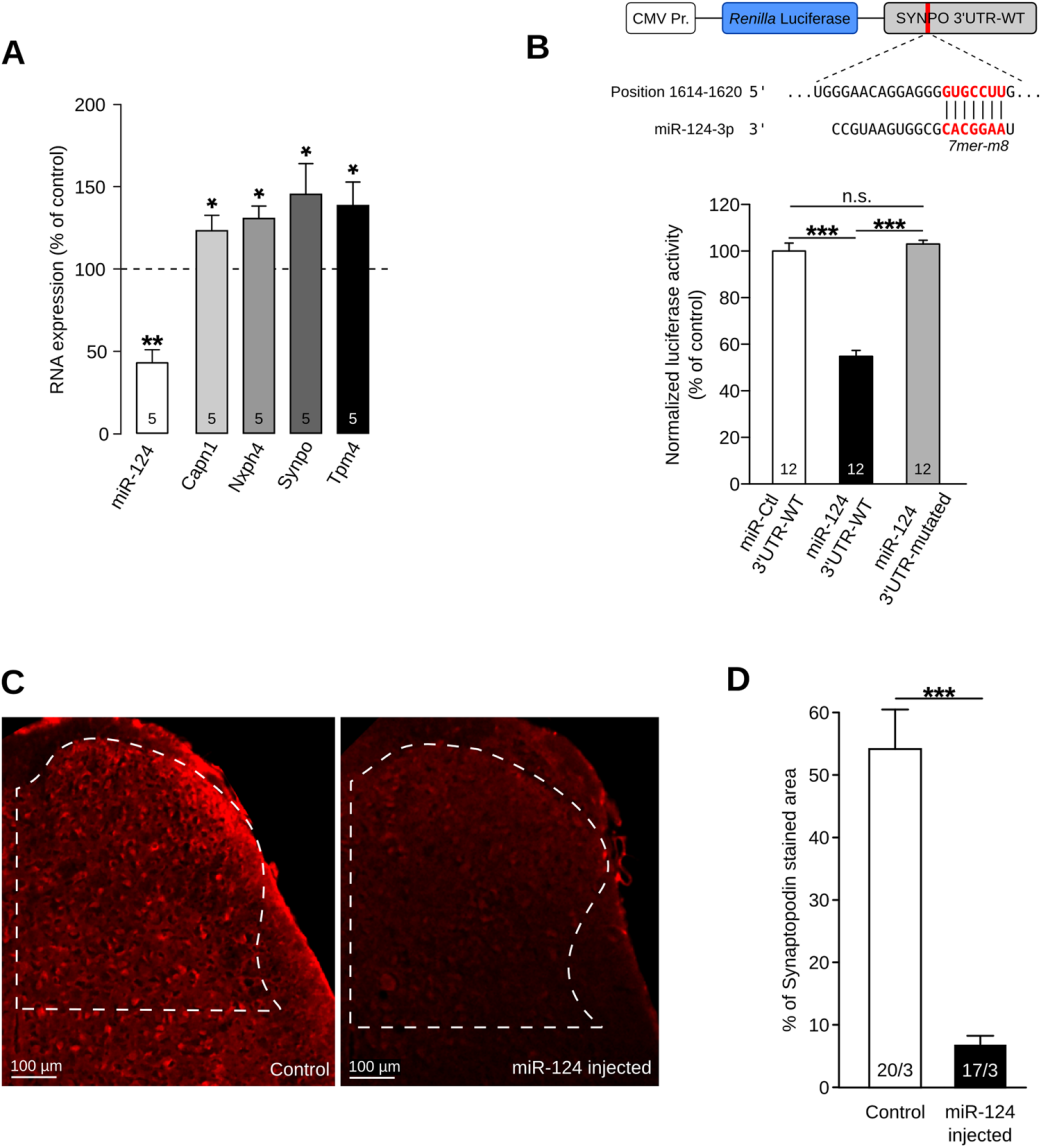
miR-124 is specific regulator of synaptopodin. (**A**) Down-regulation of miR-124 expression in cancer mice (**P < 0.01, Student’s t test) and over-expression of different predicted target genes: Capn1, Nxph4, Synpo, and Tpm4 (*P < 0.05, Mann-Whitney). (**B**) To test Synpo-miR-124 interaction, a luciferase reporter was designed by fusing Synpo 3′UTR downstream of Renilla luciferase sequence: seed region is highlighted in red. HEK-293 cells were transfected with luciferase reporter together with control miRNA (miR-Ctl) or miR-124. miR-124 induced decrease in luciferase expression compared to miR-Ctl (***P < 0.001, one-way ANOVA followed by Bonferroni post-test). To check for binding specificity, we also used a mutated 3′UTR where seed region was deleted. Mutated 3′UTR was not able to mediate luciferase regulation (***P < 0.001, one-way ANOVA followed by Bonferroni post-test). (**C and D**) Immunostaining of synpo in spinal cord after miR-124 intrathecal injections: only the dorsal horn which receive nociceptive information was quantified (white dash area). Measurement of synaptopodin stained area reveals ability of miR-124 to inhibit endogenous Synpo expression (20/3 and 17/3 denotes number of sections/animals for control and miR-124-injected mice, respectively, ***P < 0.001, Mann-Whitney).

To confirm the miRNA screening results, we used RT-qPCR to assess miR-124 expression in all animals under study. miR-124 expression level in cancerous mice was found to be strongly down-regulated (42.26% of control, Fig. 3A, P < 0.01, Student’s t test). To confirm the screening results for the 44 candidate genes, we ran RT-qPCR experiments on the whole cohort. While many genes showed a tendency to an up-regulation in cancer-pain mice without reaching statistical significance (Supp Fig. 5), only four genes appeared to be significantly up-regulated in the bone-cancer-pain condition: calpain1 (Capn1), neurexophilin4 (Nxph4), synaptopodin (Synpo), and tropomyosin4 (Tpm4) (Fig. 3A, P < 0.05, Mann-Whitney).

We next investigated the ability of miR-124 to regulate the expression of each of these four genes. To confirm miR-124 targeting predictions and to rule out the possibility that the opposite expression of miR-124 and the four candidate genes is just random, we ran a luciferase assay. This *in vitro* experiment evaluates the capacity of miR-124 to bind the 3′UTR of each candidate genes and to repress its expression. Therefore, we generated reporter plasmids by fusing the 3′UTR of Capn1, Nxph4, Synpo or Tpm4 downstream of a *Renilla* luciferase coding-sequence (Fig. 3B). The 3′UTR of Nxph4 was unable to induce a specific down-regulation of the luciferase. A moderate but significant reduction in the luciferase signal was mediated by one of the two miR-124 seed regions in Tpm4 3′UTR and by both miR-124 binding sites in Capn1 3′UTR (Supp Fig. 6, P < 0.01 and P < 0.001, respectively, one-way ANOVA followed by Bonferroni post-test). In contrast, a strong and sequence-specific reduction of luciferase activity was driven by the 3′UTR of Synpo (Fig. 3B, P < 0.001, one-way ANOVA followed by Bonferroni post-test). Thus, we demonstrated at the molecular level the ability of miR-124 to target and inhibit the translation of Capn1, Tpm4 and Synpo through binding their 3′UTR. Interestingly, the reduction observed with Synpo 3′UTR was by far the greatest (54.82% of control) and suggests efficient targeting of Synpo by miR-124 *in vivo*.

### miR-124 is an endogenous regulator of synaptopodin

To confirm the relevance of our *in vitro* analysis of miR-124 targeting, we tested the potential of miR-124 to regulate endogenous Synpo. To this end, we increased miR-124 expression in the spinal cord of naive mice by intrathecal injections of miR-124-mimics, accordingly to a previously published protocol^42^. Briefly, we performed 3 injections, one every second day, of either miR-124 mimics or control, sacrificed the animals and then immunostained Synpo in the spinal cord (Fig. 3C). In line with our *in vitro* results, miR-124 up-regulation induced a significant and specific down-regulation of Synpo expression (Fig. 3A and D, −47.03%, P < 0.001, Mann-Whitney). These results confirm that miR-124 is an endogenous and specific regulator of Synpo in the spinal cord.

### Synaptopodin is a key element of the nociceptive pathways

**Figure 4:**
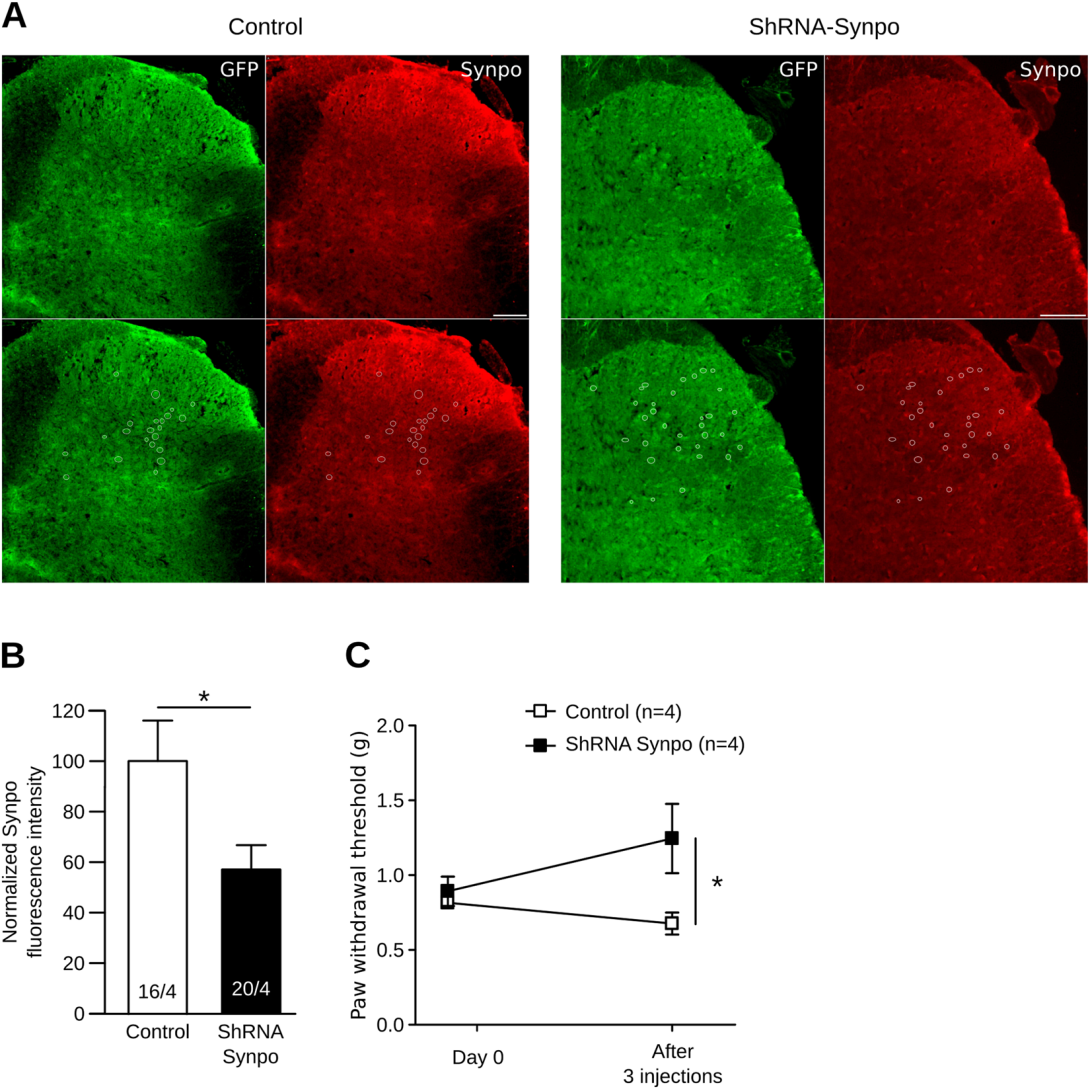
Synaptopodin is key element of nociceptive pathways. (**A**) To inhibit Synpo expression in spinal cord, we transfected a plasmid encoding both an ShRNA against Synpo and a GFP to track transfected cells; control plasmid only expressed GFP. Synpo expression assessed by immunolabeling and quantified in transfected cells only (white circles). **(B)** Quantification of Synpo immunolabeling confirmed that ShRNA strategy efficiently reduced Synpo expression by 57.15% (*P < 0.05, Mann-Whitney). (**C**) Synpo knockdown induced increase in paw withdrawal threshold as assessed with von Frey hairs (*P < 0.05, two-way ANOVA repeated measures followed by Bonferroni post-test).

To confirm the relevance of Synpo regulation in the context of bone-cancer pain, we tested whether a modification of Synpo expression has an impact on pain perception. To this end, we decreased Synpo expression in the spinal cord of naive mice by intrathecal injections of a ShRNA directed against Synpo mRNA. As for miR-124 supplementation, we performed 3 injections, one every second day. Immunolabeling of Synpo on spinal cord sections confirmed that the ShRNA strategy efficiently reduced Synpo expression by 57.15% (Fig. 4A and B, P < 0.05, Mann-Whitney). To assess the involvement of Synpo in pain perception, we measured the paw withdrawal threshold with von Frey hairs. Interestingly, mice injected with the ShRNA against Synpo showed a significant increase in paw withdrawal threshold compared to those injected with a control ShRNA, strongly suggesting that Synpo is necessary for proper pain perception (Fig. 4C, P < 0.05, two-way ANOVA repeated measures followed by Bonferroni post-test).

### miR-124 modulates bone cancer-pain by regulating synaptopodin expression

**Figure 5:**
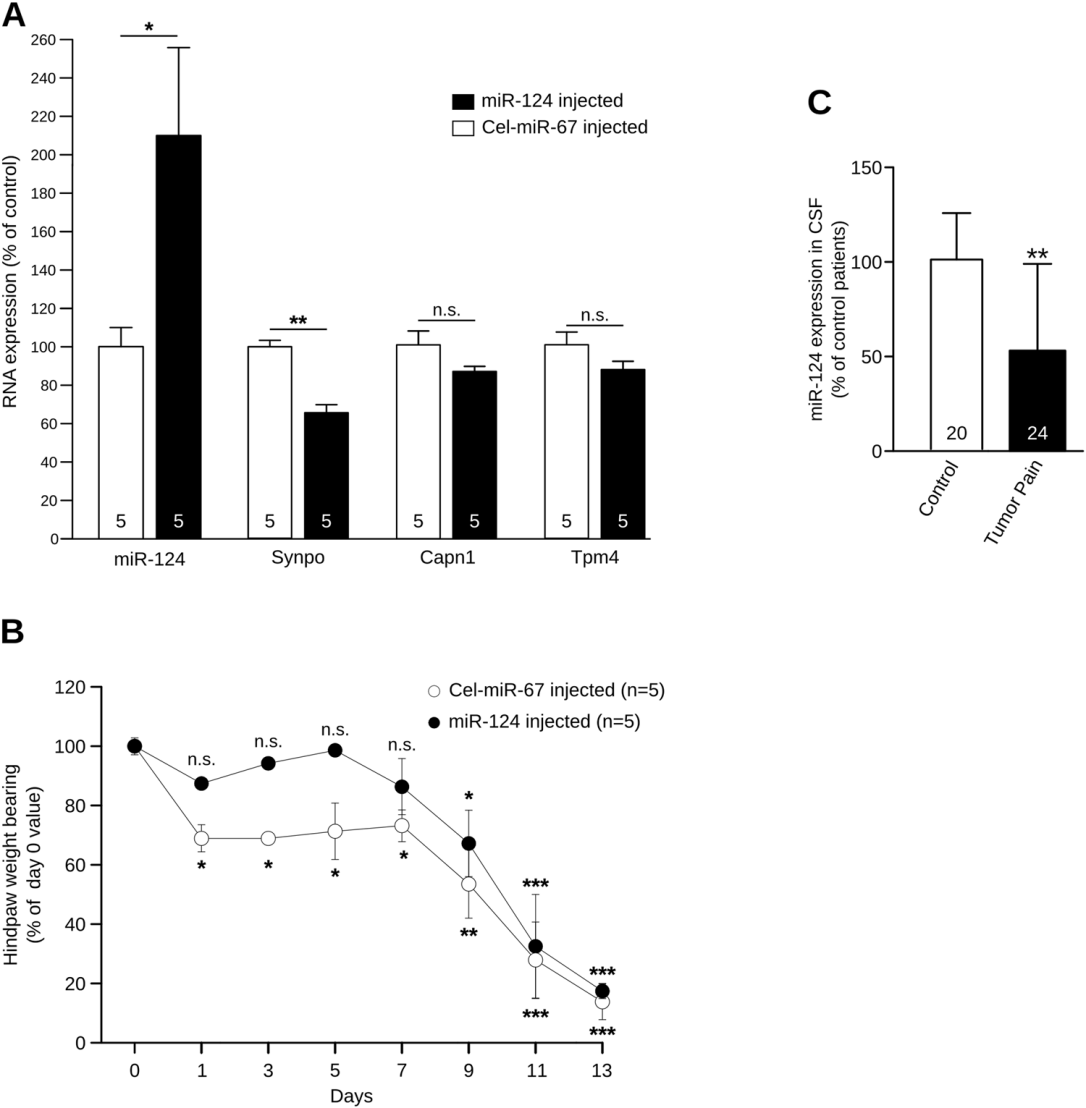
Regulation of synaptopodin by miR-124 has analgesic properties. (**A**) RT-qPCR analysis of spinal cord from intrathecally injected mice (day 14) shows up-regulation of miR-124 in miR-124 mimics-injected group (n = 5, *P < 0.05, Mann-Whitney), associated with down-regulation of Synpo when compared to Cel-miR-67-injected mice (n = 5, **P < 0.01, Mann-Whitney). (**B**) DWB measurements of cancer mice subjected to miR-124 or Cel-miR-67 intrathecal injections. Cel-miR-67 animals show an altered weight bearing in tumor-bearing hindpaw from day 1 until day 14 indicative of nociceptive state (two-way ANOVA with repeated measure followed by Bonferroni post-test, * P < 0.05, **P < 0.01, ***P < 0.001 compared to Day 0). In contrast, miR-124 alleviated nociceptive behavior in early phase of cancer pain (day 1, 3, 5 and 7, two-way ANOVA with repeated measure followed by Bonferroni post-test, *P < 0.05, ***P < 0.001 compared to Day 0). (**C**) miR-124 expression in CSF samples from bone-cancer patients (n = 24) significantly lower than in controls (n = 20) (*P < 0.05, Mann-Whitney).

To estimate the involvement of this new regulatory pathway in cancer pain mechanisms, we tested the analgesic effect of miR-124. Since miR-124 was down-regulated in the spinal cord in cancer animals, thereby making it responsible for the pain-related up-regulation of Synpo, we investigated the effect of intrathecal administration of miR-124 mimics on Synpo expression and the nociceptive behavior in cancer-pain mice. We treated bone-cancer mice (n = 5) with an intrathecal injection of 2 µg of miR-124 mimics every two days for 14 days. The control group (n = 5) which also had bone cancer was treated with a control miRNA that is expressed only in *C. elegans* (Cel-miR-67) and is predicted to have no target in mammals. As expected, RT-qPCR analysis of spinal cord tissue at day 14 showed a significant up-regulation of miR-124 in miR-124-injected mice when compared to Cel-miR-67-injected animals (Figs 5A, +113.50%, P < 0.05, Mann-Whitney). Moreover, this miR-124 up-regulation was associated with a significant down-regulation of Synpo (Fig. 5A, −35%, P < 0.01, Mann-Whitney) but with no change in Capn1 and Tpm4 expression levels (Fig. 5A). The latter result confirms that miR-124 is an endogenous regulator of Synpo. The DWB test was used to assess the nociceptive state of each group every second day until day 14 when animals were sacrificed. Cel-miR-67 animals displayed an altered weight bearing in the tumor-bearing hindpaw from day 1 (Fig. 5B, −31%, P < 0.05, two-way ANOVA repeated measures followed by Bonferroni post-test) until day 14 (−87.79%, P < 0.001, two-way ANOVA repeated measures followed by Bonferroni post-test), indicating a nociceptive state. In contrast, intrathecal administration of miR-124 alleviated nociceptive behavior since the weight borne by miR-124-injected mice was not significantly altered until day 9 of the experiment (Fig. 5B). Thus, miR-124 has a protective effect against cancer pain, by normalizing Synpo expression. Unfortunately, miR-124 injections were not able to compensate the severe nociceptive state of terminal-cancer mice between day 9 and 14 (P < 0.05, P < 0.001, two-way ANOVA repeated measures followed by Bonferroni post-test). However, these results suggest that miR-124 regulates Synpo expression in the spinal cord of mice and plays a causal role in bone-cancer pain. These effects are solely due to the analgesic effect of miR-124 and not to its anti-tumor effect. Indeed, we used a miR-124 supplementation technique which constrained the over-expression of miR-124 to the spinal cord, a method used previously to restrict miRNA expression to the spinal cord thanks to the blood-brain barrier^42^. As proof, histological analysis of the tumor in cancer mice treated with intrathecal miR-124 showed tumor growth comparable to that of non-treated cancerous mice (Supp Fig. 7).

### miR-124 measurement in patients with bone cancer pain

Since our aim was to search for miRNAs involved in bone-cancer-pain mechanisms and having a potential as therapeutic targets, we next analyzed samples from patients suffering from cancer pain. miRNAs can be extracted from cerebrospinal fluid (CSF) and are known to have a diagnostic value in neurological diseases like Alzheimer’s^43–50^. Evidence suggests that most of these miRNAs are associated with exosomes, which are extracellular vesicles released from the cells of the nervous system^51^. Thus, exosomal miRNAs in the CSF may reflect the miRNA expression in the nervous system, thereby supporting their diagnostic value in neuropathologies. Our analysis concerned two groups, one composed of 24 patients with bone cancer and suffering from strong breakthrough pain and a second group composed of 20 patients with no cancer or chronic pain as controls. We purified exosomes from patients’ CSF and quantified miR-124 expression using RT-qPCR. Interestingly, and in line with the results from the animal model, miR-124 was significantly down-regulated in the CSF of bone cancer patients who developed pain (Fig. 5C, P < 0.01, Mann-Whitney). These results provide further evidence of the involvement of miR-124 in the etiology of bone cancer pain.

## Discussion

In this paper, we addressed the role of spinal cord miRNA expression in bone-cancer pain mechanisms. A previous study revealed the involvement of miRNAs in cancer-pain mechanisms in the DRG^26^. In their work, Bali *et al*., identified 6 miRNAs dysregulated in the primary sensory neurons under cancer-pain conditions. To confirm their causal role in cancer pain and identify their targets, the authors specifically inhibited these miRNAs in tumor-bearing animals. Behavioral and mRNA-target analyses showed miR-1a-3p and chloride channel 3 (*Clcn3*) to be the most relevant miRNA-mRNA pair. Specific *Clcn3* knock-down in DRG neurons confirmed its crucial role in tumor-induced mechanical hypersensitivity. Thus, Bali *et al*. demonstrated that miRNA regulation in the DRG plays a crucial role in cancer pain mechanisms. However, they did not address the role of miRNA regulation in the spinal cord. We previously showed in neuropathic pain condition that miRNA regulation in spinal cord neurons can have a strong impact on hyperalgesia and allodynia^42^. Indeed, spinal cord neurons play a crucial role since they are the first relay in the pain pathways between the nociceptors and the supra-spinal areas which integrate pain perception.

Here, we used the murine model of bone-cancer pain developed by Schwei *et al*.^6^ which consists in injecting osteolytic NCTC 2472 sarcoma cells into the intramedullary space of the mouse femur. Histological and 3D high-resolution X-Ray (µCT) analyses confirmed the development of bone tumor. The extent of bone destruction was comparable to that obtained in the princeps study^6^ and in studies using the µCT method^52, 53^. In addition, DWB and the Hargreaves test revealed painful behavior such as mechanical allodynia and thermal hyperalgesia. Then, we screened mRNA and miRNA expression in the dorsal horn of the spinal cord to identify miRNAs and their mRNA targets playing a role in cancer-pain mechanisms. To manage these massive amounts of data, we designed a software, GEASE, which anti-correlates miRNAs and mRNAs regarding their predictive interactions. For example, if a miRNA is up-regulated, the software analyses the expression of all its putative targets and highlights the mRNAs that are down-regulated. Owing to the multi-targeting feature of miRNAs, this analysis revealed 8670 miRNA-mRNA pairs. Targeting of multiple mRNAs by a single miRNA is a fundamental characteristic of miRNA regulation that has been conserved through evolution. This feature certainly sustains the important role of miRNAs during development where a large number of genes must be jointly regulated. Furthermore, in pathological conditions where gene expression is largely altered like cancer, multi-targeting of miRNAs is likely involved. Therefore, we needed to reduce the number of candidates so we screened the miRNA-mRNA pairs to find miRNAs that could regulate multiple mRNAs implicated in pain perception and finally selected miR-124. The first criterion for choosing miR-124 is that it is one of the most regulated miRNAs in bone-cancer-pain conditions. Second, among the miR-124 targets, mRNA screening revealed 44 genes that are involved in neuronal physiology and are up-regulated in cancer-pain conditions. We already knew that miR-124 is highly enriched in the brain^30^ and, plays a pivotal role in many physiological aspects of the nervous system, including its development^31–35^, plasticity^36, 37^ and pathology^38–40^. In addition, miR-124 is linked to cancer mechanisms since it has been identified as a tumor suppressor miRNA in osteosarcoma cells^54–56^. Indeed, miR-124 supplementation reduced tumor cell migration and invasion. In addition, it has been implicated in the establishment and progression of chronic inflammatory and neuropathic pain^41^. Willemen *et al*. showed that miR-124 plays an important role in microglia activation in pain conditions. The transition from acute to persistent hyperalgesia in LysM-GRK2 + /− mice is linked to a decreased in the expression of miR-124 in the spinal cord. In addition, intrathecal injection of miR-124 totally prevented pain from becoming chronic in these mice. As a conclusion, the authors suggested that the effect observed is due to the regulation of microglia activation by miR-124. Unfortunately, the target of miR-124 regulating microglia activation was not identified.

In the present study, we show that miR-124 is down-regulated in the spinal cord of cancerous mice and identified Synpo as a primary endogenous target of miR-124. Synpo is an actin-binding molecule known as a marker of the spine apparatus (SA)^57, 58^. The SA is an essential component of mature dendritic spines of cortical and hippocampal neurons^59, 60^. It has been shown that Synpo-containing spines differ in their functional and structural properties from neighboring spines, which do not contain Synpo^58^. Synpo-deficient animals do not form an SA and demonstrate deficits in long-term potentiation *in vitro* ^57^ and *in vivo* ^22^. These structural properties of Synpo-containing spines are related to their stronger excitatory synapses, i.e., stronger evoked AMPA-receptor (AMPA-R)-mediated excitatory postsynaptic currents^61^. Taken together, these findings raise the intriguing possibility of its critical role in synaptic transmission and, thus, in pain processing. Here, we report, for the first time the expression of Synpo in the spinal cord and its up-regulation in pain in response to miR-124 down-regulation. *In vitro*, we show that miR-124 is a sequence specific regulator of Synpo. In addition, we demonstrate that its expression level in the spinal cord regulates pain perception. Indeed, inhibiting Synpo expression with an ShRNA in naive mice induced an analgesic effect. These results suggest that the up-regulation of Synpo in response to miR-124 down-regulation is one of the mechanisms of cancer pain. In line with this hypothesis, we tested the analgesic potential of miR-124. Intrathecal injections of miR-124 induced a reduction in Synpo expression in the spinal cord and had an analgesic effect. The protective effect of miR-124 injections against the onset of cancer pain suggests its clinical potential as a preventive analgesic. This potential is reinforced by our observation that miR-124 is significantly down-regulated in the CSF of bone-cancer patients who developed pain.

Further studies will aim at defining the most effective technique for miR-124 supplementation in the spinal cord of mice as well as the optimal time course of the treatment.

Finally, it would be interesting to test the effects of miR-124 on both tumor growth and cancer pain. Indeed, previous studies have demonstrated the beneficial effect of miR-124 supplementation on tumor aggressiveness^54–56^. Thus, miR-124 could have both an anti-tumoral effect on bone cancer and an analgesic effect on bone-cancer pain. This pleiotropic effect of miR-124 might reflect a major feature of the miRNA system, the targeting of multiple mRNA by a single miRNA. In the cancer cells, miR-124 might target ROR2-mediated Wnt signaling and B7-H3, a T cell co-stimulatory molecule, to produce its anti-tumor action, while it might target Synpo to produce its analgesic effect in the spinal cord.

## Materials and Methods

### Additional methods are available in Supplementary information

#### Animal model

Experiments were performed on 5-week-old male C3H/HeOuJ mice (Charles River Laboratories, L’Arbresle Cedex, France). This strain was chosen for its histocompatibility with the NCTC 2472 tumor line which was previously shown to form lytic lesions in bone after intramedullary injection^62, 63^. Tumor-bearing animals were produced as previously described^62, 63^. Briefly, anesthesia was performed with isoflurane (Iso-Vet), followed by minimal skin incision exposing the femur plateau. A 30½-gauge needle was used to make a hole in the bone, followed by tumor cells injection using as slightly bigger needle (29-gauge) to avoid leakage of tumor cells during the injection. NCTC 2472 tumor cells (10^5^ cells) suspended in 20 μl of α minimal essential medium (αMEM) were injected into the medullary cavity of the distal femur. The control group was injected with the same volume of saline solution. Bone wax (Medline) was applied on the injection site to prevent cell leakage. To assess bone destruction, femurs were scanned in an Explore Locus SP X-Ray micro-computerized tomography device (General Electric) at an isotropic resolution of 16 μm.

#### Behavioral analysis

To evaluate the development of a nociceptive state, dynamic weight bearing and plantar test were used. The dynamic weight bearing test (Bioseb)^27^, has already been used to investigate mechanical allodynia. Measurements were taken on the day of surgery (just before the injection; day 0), day 3, day 7, day 14 and day 21 after injection. Unilateral pain was evaluated through the weight borne on the ipsilateral side compared to that on the contralateral side and front paws. In addition, the percentage of time spent on each part of the animal was evaluated. Thermal hyperalgesia was tested using a Hargreaves radiant heat apparatus, also known as the plantar test (Ugo Basile Biological Apparatus)^29^. Measurements were taken on the day of the surgery (just before the injection; day 0), day 3, day 7, day 14 and day 21 after injection. A noxious heat source was placed directly under the middle of the plantar surface of the hind paw. Paw withdrawal latency (PWL) was measured during two sessions 15 minutes apart and averaged for each ipsilateral paw in each test session. Thermal exposure was interrupted after 22 seconds (cut off) to avoid tissue damage.

#### Histological analysis

After euthanasia, mice were dissected and femurs were fixed in 4% paraformaldehyde and decalcified in DC3 rapid decalcifier solution for 8 hours before being flash-frozen. 8mm-thick cryosection were obtained. Finally, sections were stained with a routine hematoxylin-eosin-safran procedure.

#### RNA Extraction and cDNA Conversion

RNA extraction was performed using the miRCURY RNA isolation kit (Exiqon) or the miRNEASY Micro Kit (Qiagen) according to the manufacturer’s instructions. For mRNA reverse transcription, the RevertAid H Minus first-strand cDNA synthesis kit (Thermo Scientific) was used, whereas the NCode™ VILO™ miRNA cDNA Synthesis Kit (Invitrogen) was used for miRNAs.

#### mRNA and miRNA Expression Profiling and correlation analysis

For mRNA and miRNA screening, we extracted RNA from the dorsal horn of the spinal cord of 6 control mice and 5 tumor-bearing mice. RNA samples were pooled to represent four groups: (i) ipsilateral and (ii) contralateral side of the dorsal horn of the spinal cord of tumor-bearing mice and (iii) ipsilateral and (iv) contralateral side of the dorsal horn of the spinal cord of control mice. Affymetrix Mouse Gene 1.0ST was used for mRNA profiling and RMAExpress was used to normalize data and determine the expression value for each gene^64^ (http://rmaexpress.bmbolstad.com/). For each gene, the Mann-Whitney test was run to assess whether the differential expression observed between the control and the tumor group was statistically significant. The accession number for data deposited in the ArrayExpress database is E-MTAB-1289.

MiRNA profiling was performed with a miRNA qPCR panel, where each miRNA was quantified with a specific forward primer and an universal reverse primer. The miRNA qPCR panel consists in two plates and covers 742 miRNA species known to be expressed in mouse. Each miRNA was quantified in each RNA sample in triplicate (6 control and 5 tumor samples) and the mean was used to determine the miRNA expression level. Briefly, results were normalized using the ΔΔCp method with multiple reference genes (U6, RNU1A1, miR-191-5p and miR-423-5p)^65^. For each miRNA, the Mann-Whitney test was run to assess whether the differential expression observed between the control and the tumor group was statistically significant.

Finally, a correlation between mRNA and miRNA expression results was sought according to the miRNA targeting predictions. To increase the relevance of this correlation, Targetscan (http://www.targetscan.org/) and miRanda (http://www.microrna.org/microrna/home.do), two databases for miRNA target predictions, were used as a matrix for miRNA-mRNA interactions. To manage the massive amount of data generated by microarray analysis, our team has set up a comprehensive database server named GEASE (Gene Expression Analysis Software Environment), which is an extension of the standard BASE software package (version 1.2). We added a new feature to enable miRNA/target-mRNA correlation according to the respective expression values. The system associates mRNAs with targeting-miRNAs that show opposite expression levels. This analysis pipeline was validated on a couple of miRNAs and mRNAs based on the manual screening of Targescan and miRanda predictions.

#### Luciferase reporter Gene Assay

For each candidate gene, a wild type 3′UTR reporter construct was obtained by annealing 47–49 bp synthesized oligonucleotides containing the putative miR-124 binding site. For candidate genes with more than one binding site for miR-124, the regulatory effect was assessed by cloning each seed region in a separate construct. Mutated 3′UTR constructs were obtained by using the same 47–49 bp synthesized oligonucleotides, although the putative miR-124 binding site was replaced with antisense nucleotides. Annealed oligonucleotides were then ligated into a modified phRL-CMV vector (Promega) downstream from the *Renilla* luciferase reporter. For the assay, 50ng of candidate gene *Renilla* reporter or mutated form were co-transfected with 750ng of either pcDNA3.1 plasmid expressing miR-124 or empty pcDNA3.1 plasmid, and *Firefly* control plasmid (pGL3, Promega) into HEK-293T cells. The activity of both *Firefly* and *Renilla* luciferase was assessed 24 h after transfection using the Dual-Luciferase Reporter Assay System kit (Promega).

#### Intrathecal administration of miRNAs and ShRNA

To over-express miR-124, we cloned the pre-miRNA sequence of miR-124 into a plasmid. To determine cells expressing this miR-124 encoding plasmid, we added a GFP-coding sequence to the construct under the control of an IRES. Thus, miR-124 over-expressing cells also express GFP. To inhibit synaptopodin expression, we cloned a ShRNA sequence directed against synaptopodin into a plasmid. To determine cells expressing this ShRNA, we added a GFP-coding sequence to the construct under the control of an IRES. Thus, ShRNA expressing cells also expressed GFP. Two micrograms of these plasmids or the corresponding controls, were solubilized in 10 µl of i-Fect reagent (Neuromics, Edina, USA), and injected intrathecally between the L5 and L6 lumbar vertebrae every two days for a total of 3 injections, according to the manufacturer’s instructions and previously published experiments^42, 66–68^.

To test the therapeutic potential of miRNAs, miR-124 and *C. elegans-*specific Cel-miR-67 purified mature mimics were obtained from Eurogentec. Two micrograms of these miRNA mimics were solubilized in 10 µl of i-Fect reagent and injected intrathecally between the L5 and L6 lumbar vertebrae every 2 days for 14 days.

#### Immunohistochemistry

Immunodetection of synaptopodin was performed on spinal cord cryostat sections (30 µm thickness). Sections were incubated for 72 hours at 4 °C with anti-synaptopodin (1/250; Synaptic Systems) and anti-GFP (1/500; AveLabs). After rising, sections were incubated with Alexa 568-conjugated goat anti-rabbit (1/500; Molecular probes) and Alexa 488-conjugated goat anti-chicken (1/200; ThermoFisher) for 2 hours at room temperature.

Image acquisition was performed with an epifluorescent microscope AxioPlan 2 (Zeiss) and a DsRi1 camera (Nikon) using the 10x objective, and the rhodamine and FITC filters.

To quantify the percentage of the area labeled for synaptopodin, we developed a macro on image j software. On the other hand, to quantify synaptopodin fluorescence intensity, we focused only on the cells expressing the ShRNA construct as determine by the expression of GFP. Then we applied the corrected total cell fluorescent method^11^. Briefly, this method quantifies the intensity of fluorescence of each cell after subtraction of the fluorescence background and correction by the area of the ROI.

#### Cerebrospinal Fluid (CSF) Sample Collection and Process

CSF samples were collected by a lumbar puncture performed by anesthesiologists at the Institut Bergonié (Bordeaux, France) and Bordeaux University Hospital (Bordeaux, France). Patients were divided in two groups; one composed of 24 bone-cancer patients and another including 20 patients with no cancer or chronic pain as controls. To recover the total exosomal RNA from CSF samples, ExoQuick-TC™ Exosome Precipitation Solution (System Bioscience SBI) was used, according to the manufacturer’s instructions. Then RNA was purified with miRNEASY Micro Kit (Qiagen). Owing to the lack of internal reference for exosomal RNA, miR-124 expression values were normalized to the volume of CSF and then the difference between the two groups was calculated.

#### Statistics

For normally distributed data (as determined by the Shapiro normality test), differences were tested using the two-tailed Student t test and ANOVA test in the event of three or more groups. The Mann-Whitney and Kruskal-Wallis tests were used when criteria for normality were not met. Bonferroni and Dunn’s tests were used as multiple-comparison post-tests. For the behavioral analysis of the tumor and control groups at different time-points, a two-way ANOVA with repeated measures was used, followed by a Bonferroni post-test. A P-value of less than 0.05 was considered significant. For all figures in which error bars are shown, data represent the mean ± SEM.

#### Ethics

All experimental procedures followed the ethical guidelines of the International Association for the Study of Pain and were approved by the University of Bordeaux’s ethics committee (agreement no. 330110008-A).

## Electronic supplementary material

supplementary info

supp table 1

supp table 2
