# Supplementary material for: Spinal miRNA-124 regulates synaptopodin and nociception in an animal model of bone cancer pain
Source: Sci Rep. 2017 Sep 8;7:10949. doi: 10.1038/s41598-017-10224-1 (PMC5591226; doi:10.1038/s41598-017-10224-1)
Supplement: Supplementary file 1 — supplementary info [file 41598_2017_10224_MOESM1_ESM.pdf]

Spinal miRNA-124 regulates synaptopodin and nociception in an animal model of bone cancer pain

Sara Elramah[1,2], María José López-González[1,2], Matthieu Bastide[1,2], Florence Dixmérias[3], Olivier Roca-Lapirot[1,2], Anne-Cécile Wielanek-Bachelet[4], Anne Vital[5], Thierry Leste-Lasserre[6], Alexandre Brochard[6], Marc Landry[1,2], Alexandre Favereaux*[1,2].

Supplementary Figures

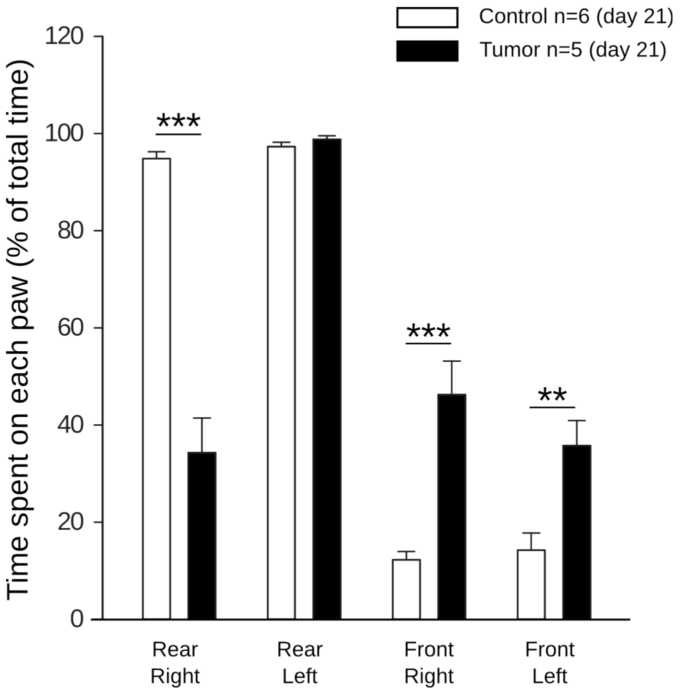

Supplementary Figure 1. Compensatory pain behavior in cancer mice.

Measurement with DWB showed a compensatory behavior in cancer mice which consists in an increase in the time spent leaning on the intact paws (** $P < 0.01$, *** $P < 0.001$, two-way ANOVA followed by a Bonferroni post-test).

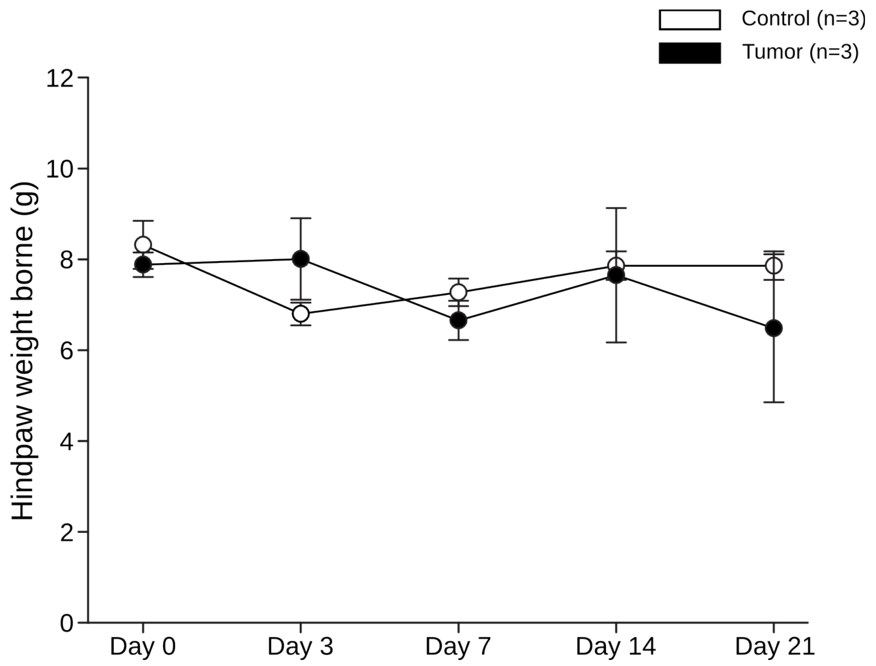

Supplementary Figure 2. Quadriceps Injection of sarcoma cells in quadriceps muscle is non-noxious and validate relevance of the cancer pain model.

Mice injected with cancer cells in the quadriceps showed no nociceptive state when compared with control group injected with saline (one-way ANOVA with repeated measures).

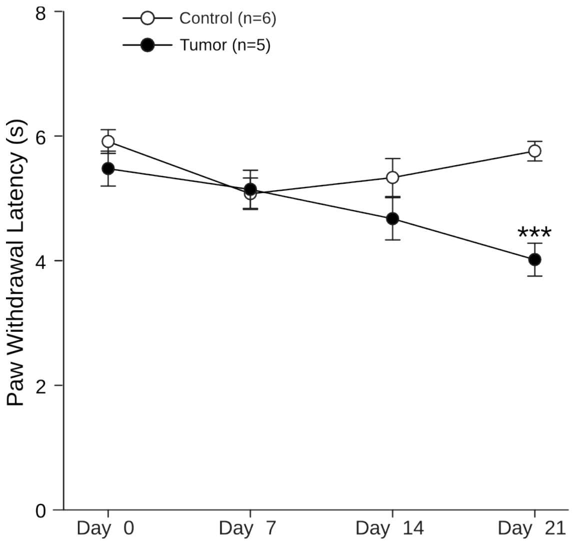

Supplementary Figure 3. Hyperalgesia behavior in cancer mice.

Testing thermal hyperalgesia using Hargreaves test revealed a significant decrease in the paw withdrawal latency of ipsilateral paws in tumor-injected group compared to controls (*** $P < 0.001$, one-way ANOVA with repeated measures followed by Bonferroni post-test).

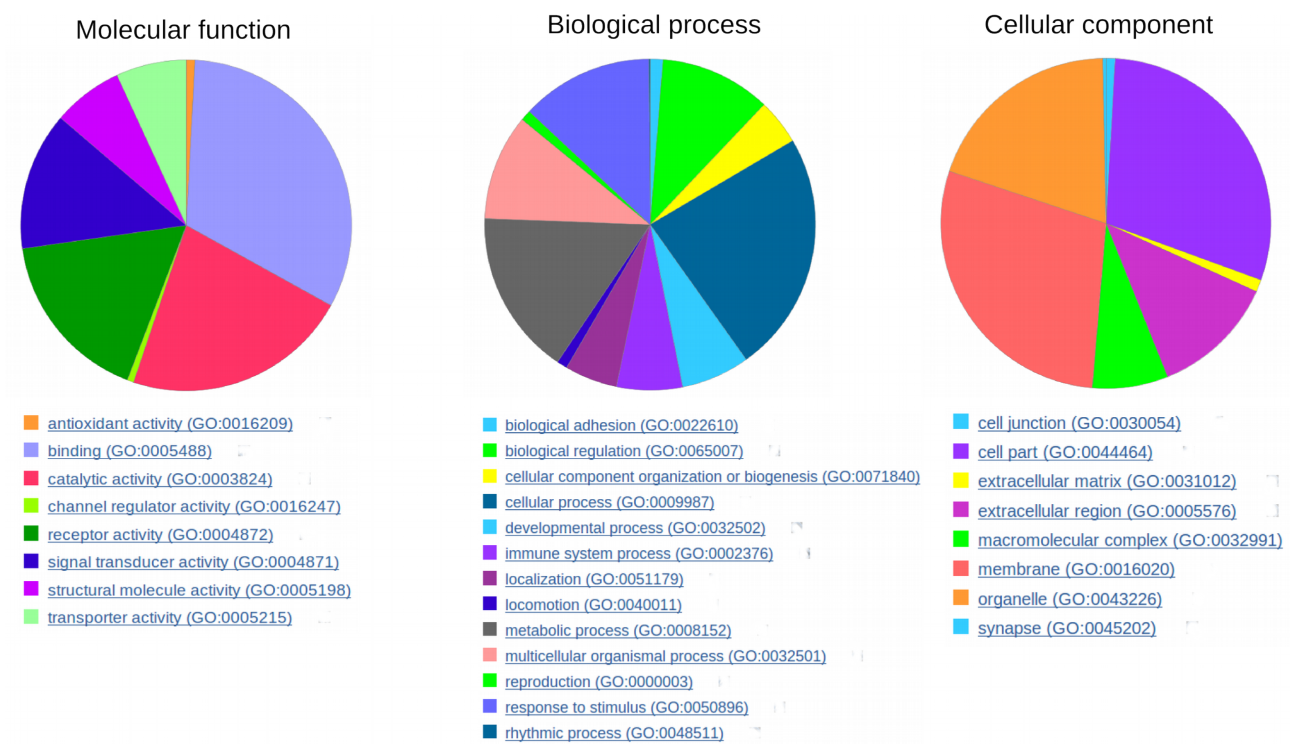

Supplementary Figure 4. Gene ontology analysis of differentially expressed mRNAs in bone cancer pain condition.

Gene expression in the dorsal horn of the spinal cord is strongly affected in bone cancer mice. GO term analysis of the differentially expressed genes shows that a wide range of molecular functions, biological processes and cellular components are affected.

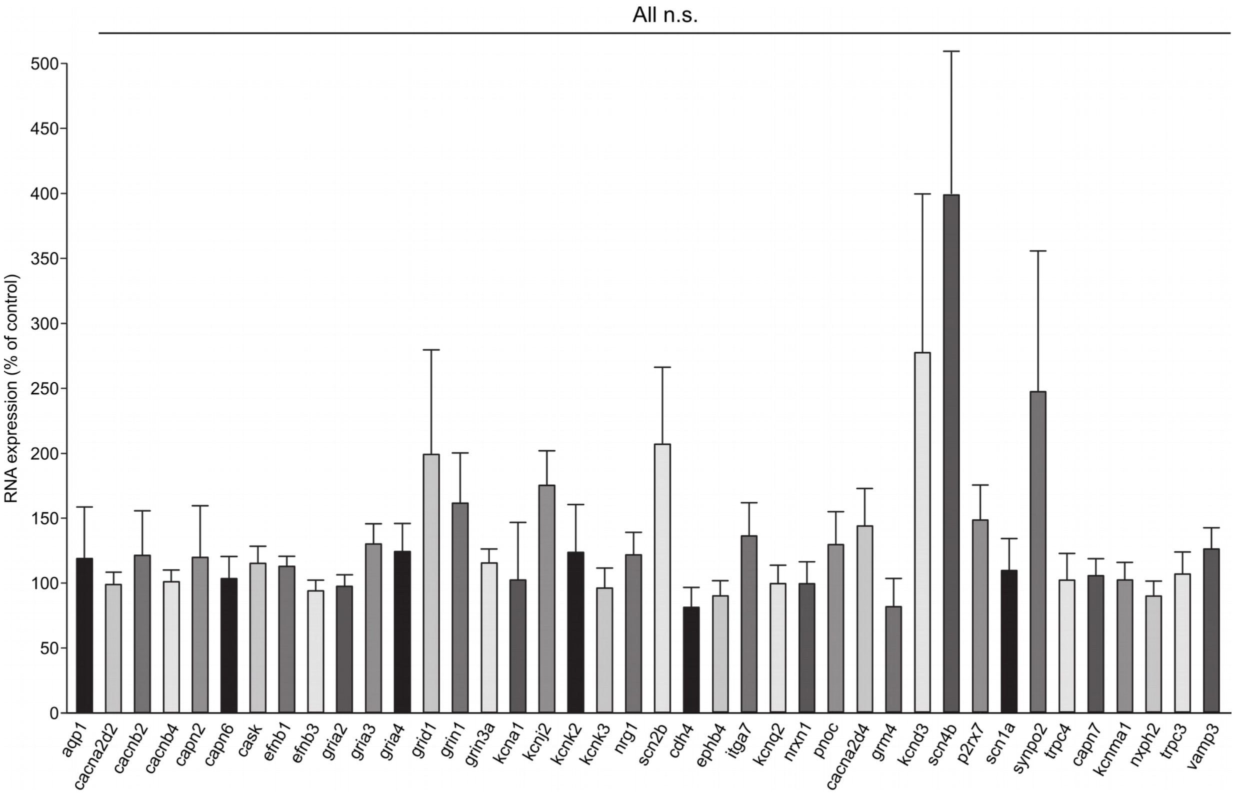

Supplementary Figure 5. RT-qPCR analysis of miR-124 target genes.

Expression analysis of a subset of predicted target genes for miR-124 known be involved in nociception. Expression of these 40 genes was not significantly affected in the spinal cord of tumor bearing mice (n=5) compared to control mice (n=6).

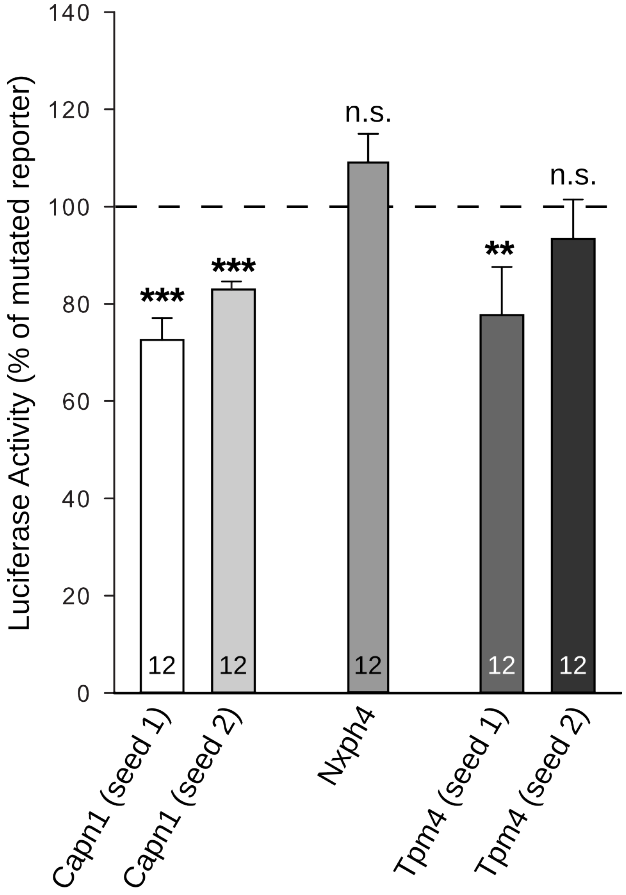

Supplementary Figure 6. Luciferase assay for Capn1, Nxph4 and Tpm4.

The 3'UTR of Nxph4 was unable to induce a specific down-regulation of the luciferase reporter, a moderate but significant reduction in the luciferase signal was mediated by one of the two miR-124 seed regions in Tpm4 3'UTR and by both miR-124 binding sites in Capn1 3'UTR (** $P < 0.01$ and *** $P < 0.001$, one-way ANOVA followed by Bonferroni post-test)

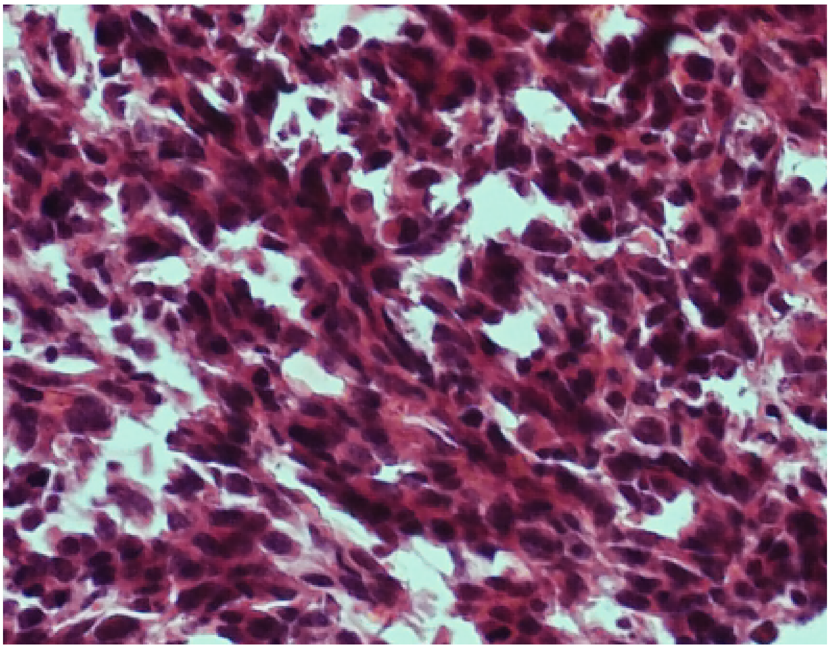

Supplementary Figure 7. Histological analysis of the tumor of miR-124 treated mice.

Cancerous mice subjected to intrathecal injections of miR-124 mimics exhibit the same tumor histology and growth as non-treated mice (Figure 1C&D).

Supplementary Materials and Methods

Osteolytic Cell Culture

NCTC 2472 tumor line (ATCC, Manassas, VA), was cultured in NCTC 135 medium (Sigma-Aldrich, St. Louis, MO) containing 10% horse serum (GIBCO).

HEK-293T Cell Culture

HEK-293T cell line was cultured in Dulbecco's Modified Eagle's Medium (DMEM) containing 10% goat serum.

Polymerase Chain Reaction and Quantitative Real-time PCR

Quantification of mRNAs and miRNAs by qRT-PCR was performed in triplicate on a Roche LightCycler 480-II using TaKaRa SYBER® Premix Ex Taq™ (Tli RNaseH Plus).

Primer sequences are the following:

succinate dehydrogenase complex, subunit A:

SDHA Forward 5' TGC-GGA-AGC-ACG-GAA-GGA-GT

SDHA Reverse 5' CTT-CTG-CTG-GCC-CTC-GAT-GG

calpain 1, (mu/I) large subunit:

Capn1 Forward 5' AGA-CCT-GGA-TGG-TGT-TGT-G

Capn1 Reverse 5' AAG-AAC-AAA-GGC-AAC-TGG-AG

neurexophilin 4:

Nxph4 Forward 5' GCC-AAG-CCC-TTC-AAA-GTC

Nxph4 Reverse 5' TGC-TCA-CTC-TGG-AAG-TTA-TAG-TC

synaptopodin:

Synpo Forward 5' GTG-AGT-CCC-ACT-TAC-AGC-AG

Synpo Reverse 5' AGA-AGG-AGG-GCT-TCC-ACA-C

tropomyosin 4:

Tpm4 Forward 5' GTA-AGT-TGG-TCA-TCC-TGG-AG

Tpm4 Reverse 5' ACC-ACA-CTT-TAG-TTC-AGA-TAC-C

To clone miR-124 in expression vector (pcDNA3.1), we amplified a 426bp genomic region containing miR-124.
